# Nanopore adaptive sampling for targeted mitochondrial genome sequencing and bloodmeal identification in hematophagous insects

**DOI:** 10.1186/s13071-023-05679-3

**Published:** 2023-02-14

**Authors:** Evan J. Kipp, Laramie L. Lindsey, Marissa S. Milstein, Cristina M. Blanco, Julia P. Baker, Christopher Faulk, Jonathan D. Oliver, Peter A. Larsen

**Affiliations:** 1 Department of Veterinary and Biomedical Sciences, University of Minnesota, St. Paul, MN USA; 2 College of Veterinary Medicine, University of Minnesota, St. Paul, MN USA; 3 Department of Animal Science, University of Minnesota, St. Paul, MN USA; 4 Division of Environmental Health Sciences, School of Public Health, University of Minnesota, Minneapolis, MN USA

**Keywords:** Culicidae, MinION, Mitochondrial genomes, Molecular barcoding, Phylogenetic capture, Tabanidae

## Abstract

**Background:**

Blood-feeding insects are important vectors for an array of zoonotic pathogens. While previous efforts toward generating molecular resources have largely focused on major vectors of global medical and veterinary importance, molecular data across a large number of hematophagous insect taxa remain limited. Advancements in long-read sequencing technologies and associated bioinformatic pipelines provide new opportunities for targeted sequencing of insect mitochondrial (mt) genomes. For engorged hematophagous insects, such technologies can be leveraged for both insect mitogenome genome assembly and identification of vertebrate blood-meal sources.

**Methods:**

We used nanopore adaptive sampling (NAS) to sequence genomic DNA from four species of field-collected, blood-engorged mosquitoes (*Aedes and Culex spp.*) and one deer fly (*Chrysops* sp.). NAS was used for bioinformatical enrichment of mtDNA reads of hematophagous insects and potential vertebrate blood-meal hosts using publically available mt genomes as references. We also performed an experimental control to compare results of traditional non-NAS nanopore sequencing to the mt genome enrichment by the NAS method.

**Results:**

Complete mitogenomes were assembled and annotated for all five species sequenced with NAS: *Aedes trivittatus, Aedes vexans*, *Culex restuans*, *Culex territans* and the deer fly, *Chrysops niger*. In comparison to data generated during our non-NAS control experiment, NAS yielded a substantially higher proportion of reference-mapped mtDNA reads, greatly streamlining downstream mitogenome assembly and annotation. The NAS-assembled mitogenomes ranged in length from 15,582 to 16,045 bp, contained between 78.1% and 79.0% A + T content and shared the anticipated arrangement of 13 protein-coding genes, two ribosomal RNAs, and 22 transfer RNAs. Maximum likelihood phylogenies were generated to further characterize each insect species. Additionally, vertebrate blood-meal analysis was successful in three samples sequenced, with mtDNA-based phylogenetic analyses revealing that blood-meal sources for *Chrysops niger*, *Culex restuans* and *Aedes trivittatus* were human, house sparrow (*Passer domesticus*) and eastern cottontail rabbit (*Sylvilagus floridanus*), respectively.

**Conclusions:**

Our findings show that NAS has dual utility to simultaneously molecularly identify hematophagous insects and their blood-meal hosts. Moreover, our data indicate NAS can facilitate a wide array of mitogenomic systematic studies through novel ‘phylogenetic capture’ methods. We conclude that the NAS approach has great potential for broadly improving genomic resources used to identify blood-feeding insects, answer phylogenetic questions and elucidate complex pathways for the transmission of vector-borne pathogens.

**Graphical Abstract:**

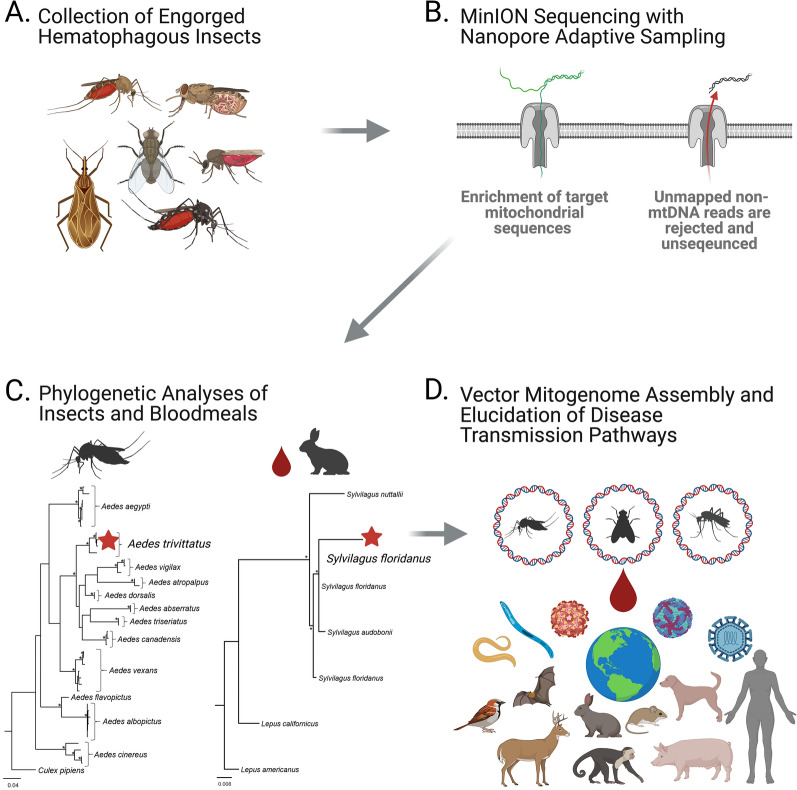

**Supplementary Information:**

The online version contains supplementary material available at 10.1186/s13071-023-05679-3.

## Background

Hematophagous insects are major disease vectors that transmit a wide variety of pathogens to their blood-meal hosts. From a One Health perspective, blood-feeding insects are responsible for considerable morbidity and mortality across global human, livestock and wildlife communities [1]. Yet, despite their global importance, vector-borne diseases remain difficult to study and adequately control. Vector-borne pathogens are often maintained in complex enzootic transmission cycles which frequently involve numerous species of both vector and vertebrate hosts. Even among groups of hematophagous insects (e.g. mosquitoes) there exists a wide diversity of disparate species which may individually exhibit substantial differences in their host feeding preferences and other natural history characteristics which impact their ability to serve as disease vectors (i.e. vector competence) [2–5].

From a broader perspective, the global diversity of hematophagous insect species presents a challenge to vector-borne disease research, as accurate species identifications are difficult in the absence of trained entomologists and region-specific taxonomic keys. For these reasons, straightforward methodologies that leverage molecular species barcoding without requiring a priori knowledge are of special interest to the global One Health community. Fortunately, the advancement of molecular tools and bioinformatic pipelines throughout the past decades has helped to inform many complex aspects of vector-borne pathogen transmission [6–9]. In particular, DNA barcoding—accomplished through PCR amplification and sequencing of select phylogenetically informative loci—has facilitated species identification efforts across hematophagous insect taxa [10, 11] and has been successfully applied to the molecular identification of vertebrate hosts from arthropod blood meals [12–15].

Across metazoan organisms, mitochondrial (mt) genes have emerged as the most popular targets for species barcoding efforts. In comparison to most nuclear genes, those encoded by mt genomes (mitogenomes) are well-suited for molecular species identification and phylogenetic reconstruction due to their relatively conserved evolutionary origins, indispensable cellular functions, predominantly uniparental inheritance and elevated mutation rates combined with very low rates of recombination [16]. Animal mitogenomes are circular in structure, relatively small in size (approx. 16 kb) and contain the same core set of 13 protein-coding genes (PCGs), 22 transfer RNAs (tRNAs) and two ribosomal RNAs (rRNAs) [16, 17]. Furthermore, mitogenomes are present at many copies within each cell, making them convenient targets for molecular analyses using a variety of biological sample types [17–23]. Sequence data of numerous mt loci (e.g. those encoding cytochrome* c* oxidase subunit 1 [COI or cox1], cytochrome* b* [cyt-b] and mtDNA D-loop) are well-documented for their phylogenetic and phylogeographic utility, having variable rates of evolution that can be leveraged to test taxonomic hypotheses and elucidate evolutionary histories [10, 11, 15, 24, 25]. The majority of barcoding efforts, including those focused on blood-feeding insects and blood-meal analysis, have leveraged traditional PCR followed by Sanger sequencing of either the COI and cyt-b genes [10, 11, 15, 26–30]. In particular, the Barcoding of Life Initiative has identified the COI gene as an ideal locus for the global standardization of sequence-based animal species identification [31]. Although such molecular barcoding has proved useful for identifying particular taxonomic lineages, it remains limited in its generation of singular-gene sequence data and potential for negative results due to PCR failure.

The weaknesses of PCR-based species barcoding and blood-meal analysis are largely overcome using de novo next-generation metagenomic sequencing approaches. Previously, high-throughput applications (i.e. Illumina sequencing) have been shown to provide the ample depth-of-coverage required for de novo taxonomic classification of blood-meal hosts [26, 32, 33]. Despite technological and bioinformatic advancements, second-generation sequencing methods (e.g. Illumina sequencing) are time-consuming, frequently require large brick-and-mortar sequencing laboratories and can be cost prohibitive. In the context of the global burden of vector-borne diseases, second-generation metagenomic approaches are largely out of reach due to their limited availability.

Nanopore sequencing technologies have increased accessibility, both financially and computationally, to genomic data. In particular, the Oxford Nanopore Technologies (ONT; Oxford, UK) MinION sequencing platform is a portable device that can be used both in laboratory and field settings for a variety of sequencing applications [34–36]. An important aspect of the MinION platform is that it sequences individual DNA or RNA molecules across a microfluidic flow cell. During a MinION experiment, single-molecule nucleotide sequences of the sequencing library are typically produced at a rate of approximately 450 bases per second, and hundreds of DNA/RNA fragments are analyzed simultaneously. Given the novelty of nanopore-based real-time sequencing, a variety of bioinformatic tools have been developed that effectively leverage the single-molecule aspect of the technology. For example, nanopore adaptive sampling (NAS) is a powerful bioinformatic method that selectively sequences individual DNA, complementary DNA (cDNA) or RNA molecules in real-time [23, 37, 38]. NAS utilizes real-time mapping against a user-specified reference file to compare nucleotides of individual DNA molecules (approx. 200–400 bp, every approx.  0.4 s) as they are being sequenced. This dynamic method has a wide number of applications and can selectively enrich DNA or RNA targets of interest (e.g. particular genes, RNA species, mitogenomes, etc.) and reject unwanted molecules during a sequencing experiment [37, 38].

**Fig. 1:**
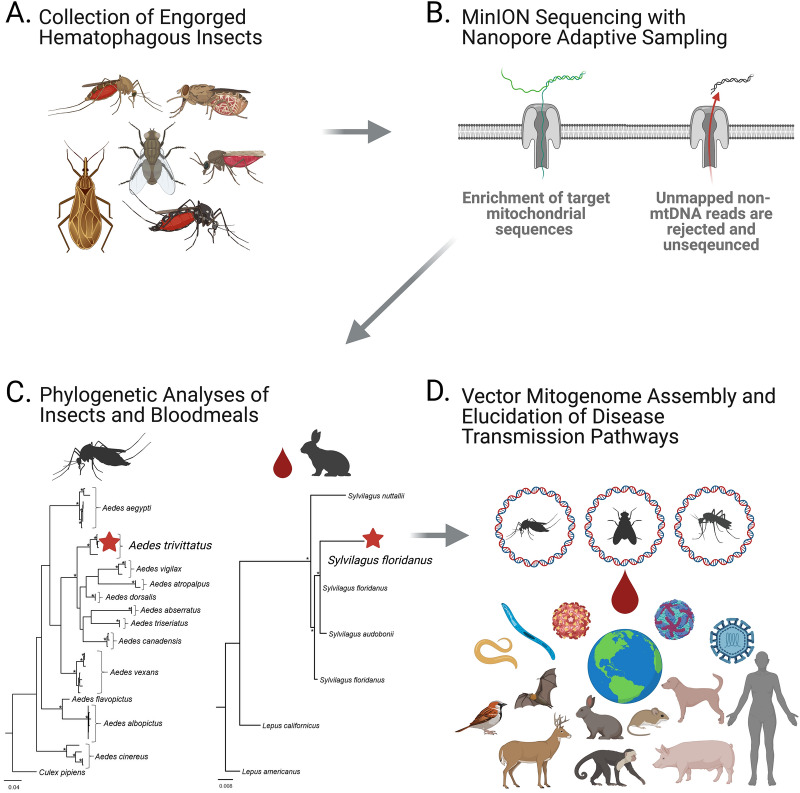
Experimental design for dual insect and blood-meal mitochondrial DNA (mtDNA) sequencing using nanopore adaptive sampling (NAS). **a** Hematophagous insects having had recent blood meals are collected from natural populations. Whole genomic DNA is extracted from individual or pooled insects. **b** Genomic DNA is prepared for sequencing on an Oxford Nanopore Technologies device (MinION platform) with access to the NAS bioinformatic pipeline. NAS is performed on blood-feeding insect DNAs with a reference file containing mtDNA sequences of congeneric or conspecific insect species and a diverse selection of putative blood-meal hosts. Exact species matches are not needed for NAS references as the method will retain sequences sharing at least 75% identity, which are suitable for guided discovery of cryptic insects and blood-meal hosts. **c** Mitochondrial sequences from NAS vector and host matches are quality filtered, and species barcoding genes (i.e. those encoding COI, cyt-b, D-loop, etc.) are used for phylogenetic analyses and species identification. **d** Complete mitogenomes are recovered from the sampled insect, thus expanding molecular resources for vector species. Recovered mt sequences of blood-meal hosts elucidate potential disease transmission pathways. Collective results of NAS experiments directly inform One Health initiatives focused on hematophagous insect biology and vector-borne disease transmission. COI, Cytochrome* c* oxidase subunit 1; cyt-b, cytochrome b; mtDNA D-loop, mt displacement loop

Combined with the long-read sequencing capabilities of the MinION platform, NAS is particularly well-suited for the selective enrichment and assembly of complete mitogenomes in animal species [23]. Moreover, NAS mitogenome sequencing can effectively capture mtDNA sequences having at least 75% identity with a given reference; thus, the method can be used for mitogenome assembly of a variety of taxa for which references are absent (Fig. 1) [23]. For these reasons the NAS method has great potential as a molecular/bioinformatic tool that can be leveraged to advance One Health research efforts focused on hematophagous insects and their blood-meal hosts. Here, we use NAS to molecularly characterize the mitogenomes of four mosquito species and a biting deer fly, and we show proof-of-concept of its utility for blood-meal analysis. We posit that NAS holds great promise for a variety of vector-borne disease research projects aimed at elucidating hematophagous insect diversity and associated pathogen transmission pathways.

## Methods

### Specimens examined

Mosquitoes were opportunistically collected using dry ice-baited CDC Miniature Light Traps (model 512; John W. Hock Company; Gainesville, FL, USA) hung approximately 6 feet above ground in Ramsey County, St. Paul, Minnesota, USA. Mosquito traps were set at 1700 hour and recovered at 1000 hour the following day. A single blood-fed *Chrysops* deer fly was opportunistically collected while conducting fieldwork within Washington County, Minnesota, USA (Afton State Park; as approved by the Minnesota Department of Natural Resources: permit 202145). All specimens were cold anesthetized and morphologically examined under a stereomicroscope. Blood-fed female mosquitoes were separated from non-target insects, and initial taxonomic identifications were based on standard morphological features [39]. For downstream nucleic acid extractions and sequencing experiments, female mosquito specimens were visually inspected to ensure that they appeared fully engorged and free of visible eggs, which suggested that all mosquitoes had obtained their blood meals within roughly 24 h of specimen collection. All specimens were submerged in RNAlater (Sigma-Aldrich, St. Louis, MO, USA) and subsequently preserved at – 80 °C prior to nucleic acid extraction and molecular analyses. A list of the specimens examined is provided in Additional file 1: Table S1.

### DNA extraction and ONT library preparation

Genomic DNA was individually extracted from a small subset of blood-fed *Culex* and *Aedes* mosquitoes, including *Culex restuans (n* = 3), *Culex territans* (*n* = 1), *Aedes vexans* (*n* = 1) and *Aedes trivittatus* (*n* = 1), and from one deer fly (*Chrysops niger*; *n* = 1), using a Qiagen DNeasy Blood and Tissue Kit following manufacturer’s instructions (Qiagen, Hilden, Germany). The resulting extracts were quantified using a Qubit 4 Fluorometer (Invitrogen, Thermo Fisher Scientific, Waltham, MA, USA). Genomic DNA libraries for ONT sequencing were produced for each specimen using Sequencing Ligation Kits SQK-LSK109 and SQK-LSK110, following standard ONT protocol. For each sample, 0.3–1.5 μg of initial DNA was used for ONT sequencing library construction. Samples were barcoded using the ONT kit EXP-NBD104, pooled, and five libraries were sequenced until completion for approximately  22–48 h on a MinION Mk1b device using R9.4 flow cells. All sequencing was performed on a Linux desktop computer with the following specifications: Intel C600/X79 series i9-10920X 12 core; Linux 5.4.0–77-generic x86_64; Ubuntu 18.04; Nvidia Quadro RTX 4000 GPU. The five sequencing experiments are denoted here as follows: A: *C. niger* NAS (2 barcodes, with 1 barcoded sample consisting of midges not included in the present study); B: *Cx. restuans* and *Cx. territans* barcoded NAS; C: *Ae. vexans* NAS (no barcode); D: *Cx. restuans* unenriched control sequencing (no barcode and no NAS); and E: *Cx. restuans* and *Ae. trivittatus* barcoded NAS (2 additional barcoded samples not included in the present study) (see Table 1).

**Table 1:** Individual nanopore sequencing experiments conducted to sequence genomic DNA from five blood-fed insects

Experiment ID	NAS technique	Species	Run time	Total bases	Number of reads (in millions)	Average read length (N50) per experiment (in bp)^a^
A^b^	Enriched using user-generated reference file	*Chrysops niger*	22 h 42 m	600 Mb	1.5	401
B^b^	Enriched using user-generated reference file	*Culex restuans*	45 h 47 m	1 Gb	2.5	462
B^b^	Enriched using user-generated reference file	*Culex territans*	45 h 47 m	2 Gb	4.5	462
C^b^	Enriched using mitogenome database as reference	*Aedes vexans*	21 h 7 m	4 Gb	10.8	536
D	Control run: no NAS	*Culex restuans*	44 h 58 m	5 Gb	6.2	1960
E	Enriched using user-generated reference file	*Culex restuans*	27 h 15 m	350 Mb	1.0	347
E^b^	Enriched using user-generated reference file	*Aedes trivittatus*	27 h 15 m	200 Mb	0.6	347

*NAS* Nanopore adaptive sampling

NCBI accession numbers for NAS enrichment files are provided in Additional file 2: Table S2

^a^N50 metric: sequence length of the shortest contig at 50% of the total assembly length

^b^Data from these experiments were used for downstream insect mitogenome assemblies

### Basecalling

Sequencing was initiated using the MinKNOW GUI, v4.3.20, software (ONT) in which adaptive sampling is integrated. Reference files containing publicly available mitogenome sequences and select barcoding genes (i.e. those encoding COI, cyt-b) were compiled in FASTA format and used for NAS-based enrichment. For NAS sequencing experiments A (*C. niger*), B (*Cx. restuans* and *Cx. territans*) and E (*Cx. restuans* and *Ae. trivittatus*), we manually curated a reference FASTA containing mitogenome assemblies of related (i.e. genus level) blood-feeding insects, as well as an assortment of mammalian and avian mitogenomes based on species considered to be potential blood-meal hosts for our study location (a list of GenBank accession numbers is provided in Additional file 2: Table S2). For NAS experiment C (*Ae. vexans*), we elected to use the NCBI RefSeq download of the complete mt genome database (downloaded 16 Sept 2021). Real-time basecalling was performed using the FAST basecalling model in Guppy and mapped to a provided reference file using the NAS. After completion of the sequencing runs, post-hoc high-accuracy basecalling was performed using Guppy (v5.0.11; release with GPU-enabled basecalling for Ubuntu) prior to downstream analyses. The NAS software pipeline provides an optional immediate readout of reads mapping to particular reference sequences, and real-time alignment data can be recorded in the resulting sequencing summary output text file. For experiments C and E, this optional readout was utilized to extract individual sequences based on successfully aligned reads contained in the sequencing summary output text file. For the remaining NAS experiments (A and B), targeted mtDNA reads were mapped using Minimap2 (v2.17-r941) and indexed using SAMtools (v1.9) in post-hoc bioinformatic analyses.

### NAS versus control sequencing

A control nanopore sequencing experiment (experiment D) was conducted to test the effectiveness of NAS. This experiment generated sequences from genomic DNA of a single mosquito (*Cx. restuans*) without NAS for approximately 45 h. During the sequencing runs, basecalling was accomplished using the FAST model, and post-hoc basecalling was conducted using the high-accuracy model of Guppy to keep all other sequencing variables (other than NAS) equivalent. Sequences from experiment D were filtered based on a quality score of 10 and read lengths between 1 and 16 kb using NanoFilt v2.7.1 [40]. Filtered reads were subsampled at random using seqtk v1.3 (https://github.com/lh3/seqtk) and used in comparison analyses. Randomly sampled reads were mapped to the *Cx. pipiens* mitogenome using the program Minimap2 v2.17 [41] and the percentage of assembled reads were noted. Experiment D was compared to NAS experiment B for *Cx. restuans* and *Cx. territans*, using the same filter parameters, random subsampling technique and mapping program.

### Mitogenome assemblies and phylogenetics

Reads were filtered for mitogenome assembly using NanoFilt with a minimum Q-score of 10 and read lengths of between 1 and 16 kb [40]. Filtered reads for individual samples were de novo assembled using Flye v2.8.3 following methods outlined in Wanner et al. [23]. Subsequent mitogenome assemblies were polished to produce more accurate assemblies using Medaka v1.4.3 (https://github.com/nanoporetech/medaka). Open reading frames (ORFs) were identified and annotated using MITOS2 web server (http://mitos2.bioinf.uni-leipzig.de/index.py) with specification for metazoan RefSeq and invertebrate genetic code [42]. Each annotated mitogenome was visually inspected using Geneious Prime software v2021.2.2, and aligned against a published and annotated mitogenome of another member in each genus. Where necessary, the start and end positions of particular genes were manually adjusted based on those of previously characterized mitogenomes. Polished and assembled mitogenomes were visualized using OGDRAW v1.3.1 (https://chlorobox.mpimp-golm.mpg.de/OGDraw.html) [43].

After annotation of the mt genomes, COI gene sequences were used in phylogenetic analyses to verify species identification. COI sequences were downloaded from GenBank for each appropriate species (*Culex*, *Aedes* and *Chrysops*) to build phylogenies. Appropriate outgroup samples also were downloaded from GenBank and included in each analysis to root the phylogeny. Sequences were aligned using MAFFT v7.475 [44], and subsequent alignments were used in a maximum likelihood analysis with the following parameters in RAxML v8.211 [45]: (i) model of evolution was set to GTRGAMMAI; and (ii) analyses were performed using 1000 bootstraps. The resulting best tree for each analysis was visualized using FigTree v1.4.1 (https://github.com/rambaut/figtree). Initial putative blood-meal identifications were determined by real-time alignment to vertebrate mt genomes during each sequencing experiment (see Additional file 2: Table S2). Following post-hoc high-accuracy basecalling, confirmation of blood-meal host identifications were achieved using Minimap2 and SAMtools to re-map reads across vertebrate mt genomes to characterize successfully mapped reads. Average coverage was calculated for the human blood meal (experiment A), and phylogenetic trees were generated (using the same parameters described above) for the house sparrow (*Passer domesticus*) and eastern cottontail (*Sylvilagus floridanus*) blood meals to determine statistical nodal support as mtDNA coverage was considerable in reads derived from these vertebrate hosts.

## Results

### Output and performance of nanopore sequencing experiments

Genomic DNA was isolated from five species of blood-fed insects (species identifications were confirmed using COI barcoding, as described below): *C. niger* (*n* = 1), *Cx. restuans* (*n* = 3), *Cx. territans* (*n* = 11), *Ae. vexans* (*n* = 1) and *Ae. trivittatus* (*n* = 1). Molecular data for each specimen was generated across five ONT sequencing runs (Table 1). The number of bases generated for each sample ranged from 200 Mb to 5 Gb, and the number of reads generated ranged from 0.6 million to 10.8 million. Average read length (N50) was lower in sequences generated from NAS runs (347–536 bp) than those generated in the control experiment (1.96 kb), as expected (Table 1). Raw FASTQ files generated for each sequencing run were submitted to the Sequence Read Archive (SRA) at NCBI (Bioproject: PRJNA775614; Biosamples: SAMN22604479-SAMN22604483; SAMN22888850-SAMN22888851).

### Comparison of NAS and control sequencing for mitogenome enrichment

Our comparison of the NAS method versus a control nanopore sequencing experiment (i.e. NAS off; experiment D) revealed that approximately 65.8% and 75% of reads in the NAS active experiment (experiment B: *Cx. territans* and *Cx. restuans*, respectively) mapped to the *Cx. pipiens* reference, whereas 1.8% of reads in the control experiment (experiment D) mapped to the same reference (Table 2). Given that experiment D consisted of a single mosquito, we performed sequence subsampling analyses and found similar percentages (NAS: > 65% reads mapped; control: approx. 2% reads mapped; Additional file 3: Table S3).

**Table 2:** Percentage of total reads mapping to a *Culex pipiens* mitochondrial genome reference (NC_015079) for nanopore adaptive sampling versus control sequencing experiments

Experiment ID	Species	Number of sequences after filtering	Number of sequences mapped	Percentage of reads mapped
D	*Culex restuans*	1,609,492	29,289	1.8
B	*Culex restuans*	9664	7247	75.0
B	*Culex territans*	18,736	12,299	65.6

Analyzed samples consisted of a single *Culex restuans* within the control ONT (Oxford Nanopore Technologies Inc) sequencing experiment (experiment D) and 2 barcoded samples (*Cx. restuans* and *Cx. territans*) from an NAS-enabled experiment (experiment B) for mitochondrial DNA enrichment

### Assembly and annotation of mt genomes

**Fig. 2:**
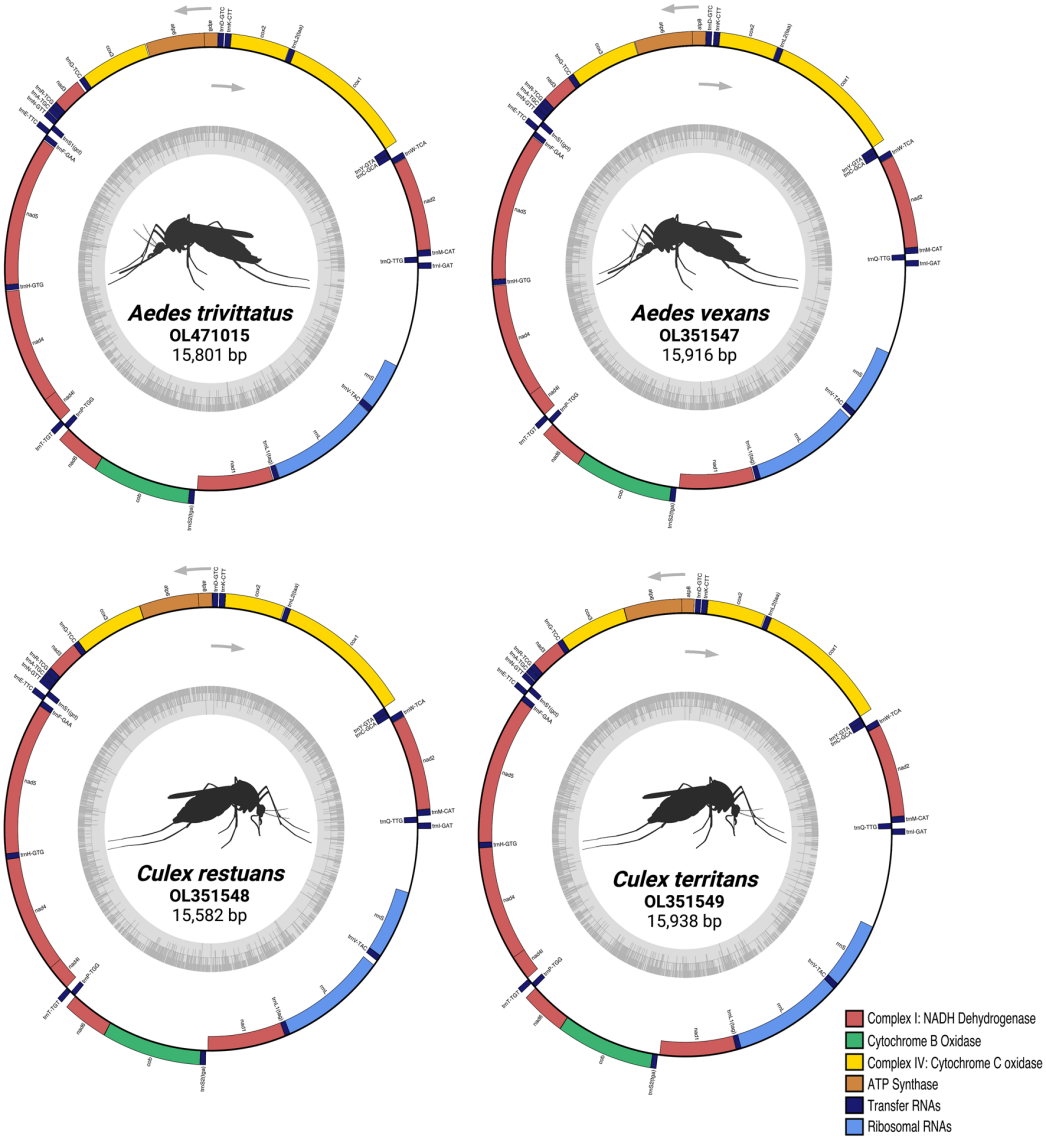
Circular mt genome maps for the four mosquitoes sequenced using nanopore adaptive sampling. The circular diagram for *Chrysops niger* is presented in Additional file 4: Figure S1. Orientation of transcription for protein coding, ribosomal RNA and transfer RNA genes is denoted by gray arrows. Genes encoded on the majority strand (transcribed counterclockwise) are shown on the outer portion of each circular diagram, while those encoded on the minority strand (transcribed clockwise) are shown on the inner portion. Relative A + T content is visualized as a percentage on the innermost circle in light gray

For each species sequenced using NAS enrichment, we assembled a single, circular contig from aligned mtDNA reads. Mean assembly coverage for each mitogenome is reported as follows: *C. niger:* 56×; *Cx. restuans*: 612×; *Cx. territans*: 1342×; *Ae. trivittatus*: 46×; *Ae. vexans*: 750×. In addition to these successfully assembled mitogenomes, we also attempted assembly using aligned reads generated during the control experiment (experiment D: *Cx. restuans*). Here, we noted that 19 contigs of high coverage (1846×–2428×) and short lengths (426 bp–958 bp) were generated during Flye assembly and this ultimately precluded our ability to obtain a high-quality mitogenome in the absence of NAS-based enrichment. The five NAS-derived mitogenomes were all of an intermediate size, ranging from 15,582 to 16,045 bp in total length (Fig. 2; Additional file 4: Figure S1). Nucleotide bias was also consistent across all five mitogenomes and ranged from 78.1% A + T to 79.0% A + T in total nucleotide content.

Annotation of our assemblies indicated that each encoded a total of 37 genes, which included the expected assemblage of PCGs (*n* = 13), tRNAs (*n* = 22) and rRNAs (*n* = 2). A short region lacking gene content (i.e. control region) at the suspected site of replication initiation was also observed in each sequenced mitogenome. Among our *Aedes* and *Culex* mitogenomes, the majority strand (J-strand) was found to encode nine PCGs and 13 tRNAs, while the minority strand (N-strand) was found to encode four PCGs, nine tRNAs and both rRNA genes. Overall, this organization of gene content as well as the specific order of genes was found to be identical among our three mosquito mitogenomes and consistent with that reported in other members of the Culicidae family [46, 47]. Our *C. niger* deer fly mitogenome differed slightly in that the J-strand encoded nine PCGs and 14 tRNAs, with the N-strand encoding the remaining four PCGs, eight tRNAs and both rRNAs. Importantly, this organization is consistent with previously sequenced mitogenomes for dipteran members of the suborder Brachycera [48]. Complete annotated mitogenome assemblies were submitted to the NCBI Organelle database (Additional file 2: Table S2).

### Molecular identification of insect species and blood meals

Genetic identification for each vector species was determined using the complete COI gene that was annotated from the assembled mitogenomes reported herein (experiments A-C) or from consensus sequences generated by mapping to a COI gene reference (experiments D and E). The resulting best trees generated from a bootstrapped maximum likelihood analysis are reported in Additional file 5: Figure S2. Each vector COI sequence generated for this study was statistically supported and formed a monophyletic clade of the appropriate species.

**Fig. 3:**
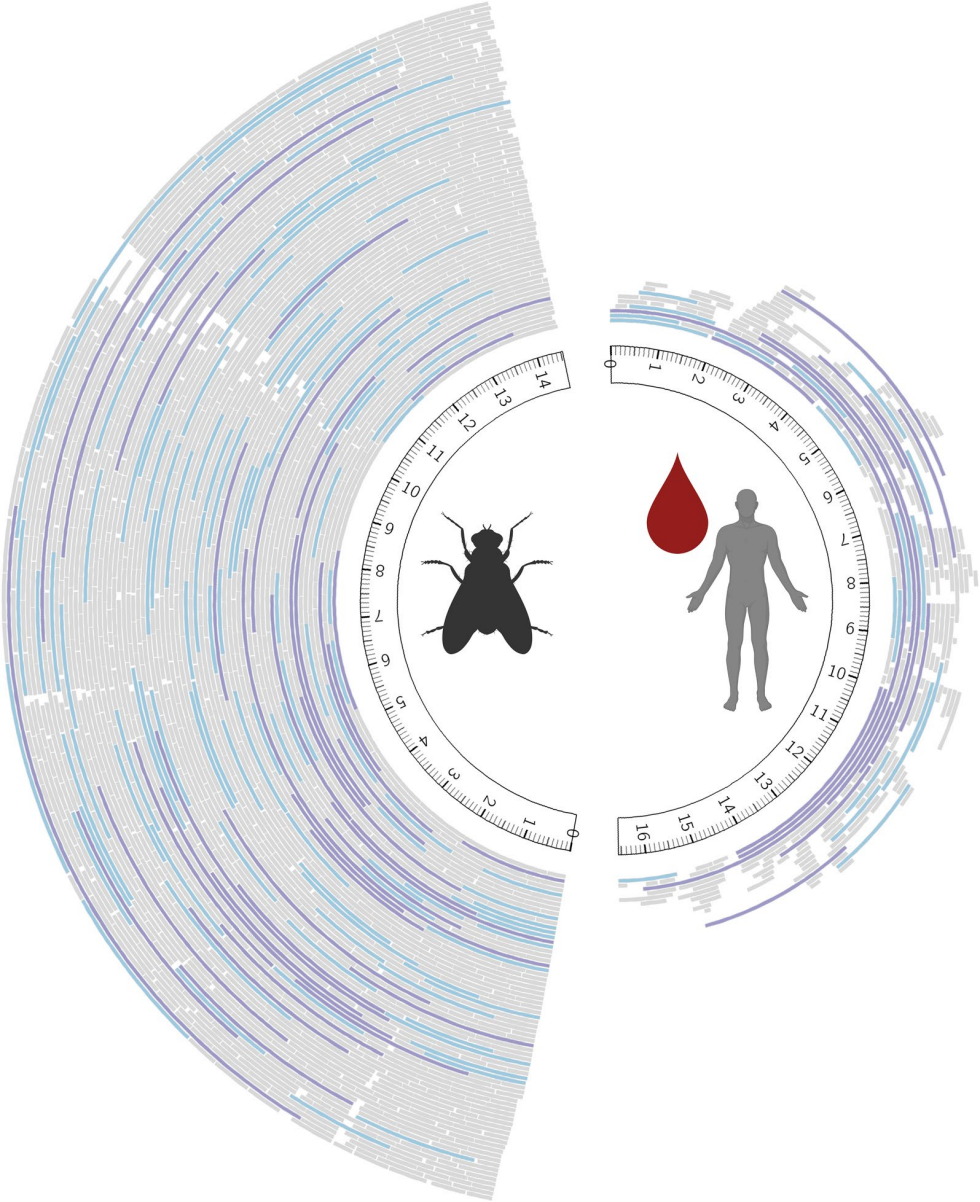
Circos diagram showing nanopore adaptive sampling reference-guided mitogenome mapping results for a Minnesota black deer fly *Chrysops niger* (left; reference *Chrysops silvifacies* from China NCBI KT225292.1) and its human blood meal (right; reference human mitogenome NCBI NC_012920.1). Outer layers depict individual ONT reads mapping to reference mitogenomes where reads > 2 kb are identified in purple, reads with length 1-2 kb in blue and reads < 1 kb in gray. Reference mitogenomes for *C. silvifacies* and human are identified on the left and right, respectively, with labeled major tick marks every 1 kb and unlabeled minor ticks every 100 bp. ONT, Oxford Nanopore Technologies Inc

**Fig. 4:**
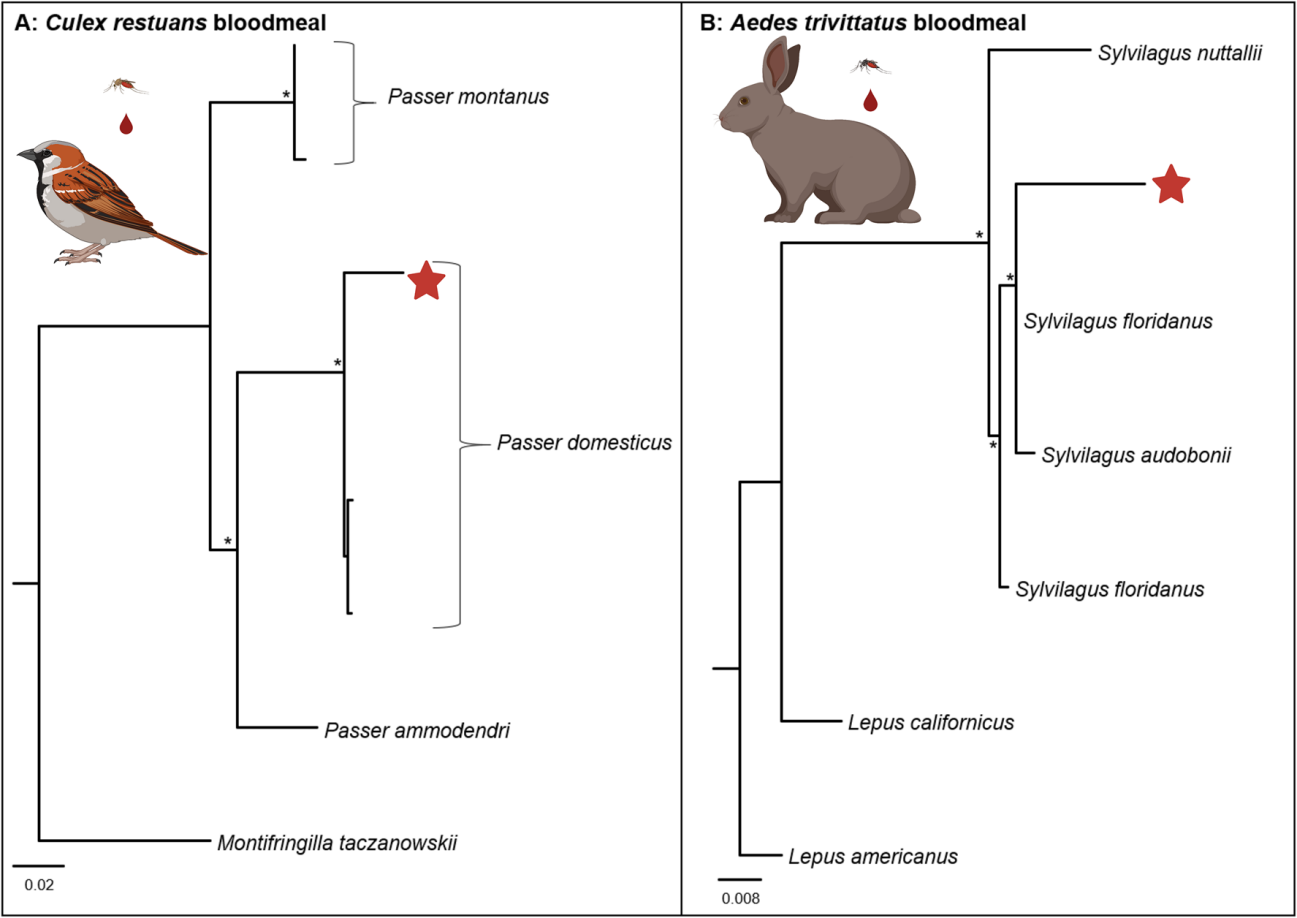
Phylogenetic trees constructed of *Culex restuans* and *Aedes trivittatus* blood meals. Barcoded sequences generated from *Cx. restuans* and *Ae. trivittatus* (experiment E) are denoted by a red star, and statistically supported nodes (≥ 75) are denoted with a single asterisk (*). Genbank accession numbers of sequences included in both phylogenetic analyses are provided in Additional file 6: Table S4. **a** Maximum likelihood analysis with 1000 bootstrap replicates for the control region (i.e. D-loop) of Passerine birds. **b** Maximum likelihood analysis with 1000 bootstrap replicates for the 12S gene of rabbits (genera *Lepus* and *Sylvilagus*)

A blood meal identified as human was successfully sequenced from *C. niger* (experiment A). We recovered over 200 mtDNA sequences of the human blood meal, and mapped reads primarily ranged from approximately 100 to approximately  8 kb in length, with a single read spanning nearly the entire human mitogenome (16,123 bp; Fig. 3). Experiment E generated blood-meal sequencing reads for two vector species. For the *Cx. restuans* vector, 16 reads mapped to the house sparrow (*P. domesticus*) mitogenome, ranging in length from 106 to 2475 bp. Of these 16 mapped reads, three reads mapped to the control region (CR) of the house sparrow mt genome. A maximum likelihood analysis revealed that the sequences generated from the CR formed a statistically supported clade with *P. domesticus* (1.0% divergence within the *P*. *domesticus* clade; Fig. 4a; Additional file 6: Table S4). The *Ae. trivittatus* vector from experiment E resulted in a blood-meal host identified as an eastern cottontail rabbit (*S. floridanus*) with a single read (955 bases long) mapping to the 12S gene. A phylogenetic analysis of the 12S gene supports the molecular identification of the blood meal to *S. floridanus* (2.0% divergence within the *S*. *floridanus* clade; Fig. 4b).

## Discussion

In the present study we demonstrate the dual utility of NAS for mitogenome assembly and blood-meal identification in hematophagous insects. Using data generated through NAS, we successfully enriched mtDNA, and assembled and annotated entire mt genomes of four mosquito species (*Ae. vexans*, *Ae. trivittatus*, *Cx. restuans*, *Cx. territans*) and the black deer fly (*C. niger*) (Fig. 2). Of these, the mt genome assemblies for *Ae. trivittatus*, *Cx. restuans*, *Cx. territans*, and *C. niger* are the first to become available for the respective species. Using a meta-mt barcoding approach, we confirmed the vertebrate sources of the blood meals of *Cx. restuans* (house sparrow), *Ae. trivittatus* (eastern cottontail rabbit) and *C. niger* (human) (Figs. 3, 4; experiments A and E).

NAS is a relatively new bioinformatic tool [38], thus comparisons to traditional nanopore sequencing runs are essential. The analyses presented herein and those of others [23, 37, 38, 49, 50] clearly show that NAS consistently enriches reference sequences (herein, mt sequence data) at levels ranging from 0.96-fold to 5.4-fold versus control ONT experiments. The NAS experiments conducted herein successfully captured entire mitogenomes of the hematophagous insects, with coverages ranging from 56× (*C. niger*) to 1342× (*Cx. territans*). Moreover, the mitogenome of the control run (experiment D; NAS off; *Cx. restuans*) was not fully assembled into a single, circular contig. This finding is interesting given we secured an estimated coverage of over 23,000 sequences from the control experiment (based on reference-guided mapping to the *Cx. pipiens* mt genome). In light of our failure to assemble a complete circular contig from these control data, we posited that there were likely numerous nuclear mtDNA segments (NUMTs) that survived filtering, ultimately resulting in the generation of 19 short, linear contigs during the Flye assembly process. To evaluate this further, we subsampled these mapped approximately 23,000 reads from the control run (experiment D) and characterized them using BLAST (Basic Local Alignment Search Tool) against the NCBI nucleotide database. For a majority of these subsampled reads, we observed BLAST matches to *Culex* mtDNA which consisted of only a short percentage (approx. 5–10%) of the overall read query. Further analysis of the flanking regions on these subsampled reads most closely matched against nuclear regions of *Cx. pipiens pallens* (NCBI assembly accession: GCA_016801865.1) and *Cx. quinquefasciatus* (NCBI assembly accession: GCA_015732765.1) whole-genome assemblies, suggesting that these reads did indeed contain NUMTs with mt pseudogenes. NUMTs are common across eukaryotes and have been reported in a variety of mosquito taxa [46, 51, 52]. Our findings suggest that targeting mtDNA using NAS—which bases its enrichment through reference-based mapping of the initial 200–400 bp for each read sequenced—may help resolve the issue of inadvertent NUMT capture, as NUMTs contained within long stretches of nuclear DNA would not map against our mtDNA references and be rejected from complete sequencing using our approach. Thus, NAS can work to streamline sequencing and assembly for mitogenomes while avoiding the confounding effects of NUMTs that remain challenging with traditional molecular methods.

Similar to Wanner et al. [23], the results reported herein clearly document the utility of NAS for the enrichment and downstream assembly of complete mt genomes. In light of these observations, we posit that NAS will be a particularly useful tool for a multitude of studies, ranging from the inter- and intra-species characterization of mitogenome variation to biodiversity assessments of a wide variety of taxa wherein mt-based species barcoding is routinely conducted [31]. For hematophagous insects in particular, taxonomic identification can be challenging for both novice and skilled entomologists using morphological characters alone, especially for cryptic species. Moreover, given the range of expansions associated with climate change and anthropogenic-driven introductions, traditional morphological keys for a given geographic area may be unable to resolve species status for resident hematophagous insects. For these reasons, a robust molecular-based approach that facilitates the taxonomic identification of blood-feeding insects will greatly aid vector-borne disease research and vector biosurveillance. In the present study, we demonstrate how our NAS approach will directly contribute to such efforts by beginning with unidentified field-collected specimens and successfully producing complete mitogenome assemblies and molecular identifications for our sampled species. We highlight the natural history of the insects sampled herein to demonstrate the power of our end-to-end NAS mtDNA approach.

*Aedes vexans* is a cosmopolitan species and often represents the most common mosquito species collected in the Upper Midwest, USA [53]. It is considered a competent vector of a variety of pathogens, including West Nile virus, Eastern and Western encephalitis virus, Zika virus, St. Louis encephalitis virus, Rift Valley fever virus and *Dirofilaria immitis*, although is rarely considered a species of significant disease concern [54–56]. *Aedes trivittatus* shares many ecological characteristics with *Ae. vexans* and is an abundant summer floodwater mosquito throughout the American Midwest; *Ae. trivittatus* appears to feed chiefly on mammals, including the eastern cottontail rabbit (*S. floridanus*)*,* [57–59], a feeding preference which is supported by our blood-meal analysis findings. Similar to *Ae. vexans*, *Ae. trivittatus* is likely a competent vector of both West Nile virus [60] and *D. immitis* [61], although its precise role in pathogen transmission in North America has not been well-characterized. The *Ae. trivittatus* mt genome assembly provided herein and deposited into NCBI (accession number OL471015) is the first to be reported for this species. *Culex restuans* is distributed throughout the USA and Canada [39, 62]; it is increasingly being recognized as an important vector of West Nile virus, particularly in urban and suburban areas [63], and is likely capable of transmitting St. Louis encephalitis virus [64]. Notably, adult female *Cx. restuans* mosquitoes are largely indistinguishable from North America's primary West Nile virus vector, *Cx. pipiens*, based on external morphological features [65, 66]. The *Cx. restuans* mt genome assembly provided herein (accession number OL351548) is the first to be reported for this species and represents an important resource for the molecular differentiation of these medically important West Nile virus vectors. *Culex territans* is widespread in the Eastern USA where it feeds on frogs [67]. It transmits a hepatozoon parasite to frogs but is unlikely to serve a major role in mammalian disease transmission [68]. The *Cx. territans* mt genome assembly provided herein and deposited on NCBI (accession number OL351549) is the first reported for this species. The deer fly *C. niger* is widely distributed in Eastern USA and south-eastern Canada and is associated with marshy areas where its larvae feed on organic matter in soil [69]. It is a common pest of livestock [70]. Other members of the genus are involved in the mechanical transmission of *Fransicella tularensis* in Western USA [71]. The *C. niger* mt genome assembly provided herein and deposited on NCBI (accession number OL351550) is the first to be reported for this species.

Regarding our NAS-based recovery of the mitogenomes of the *C. niger* deer fly, the only available mt genome for reference within the genus was from *C. silvifacies* collected from China. Our de novo assembly of the *C. niger* mitogenome is separated by a genetic distance of approximately 9.4% Kimura-2 Parameter values from *C. silvifacies.* Likewise, we used the mt genome for *Cx. pipiens* collected from Tunisia as reference for our *Cx. restuans* and *Cx. territans* NAS sequencing. The de novo assembled mitogenomes generated herein for *Cx. restuans* and *Cx. territans* have a genetic distance of approximately 5.5% and approximately 8.0% from *Cx. pipiens*, respectively. These results show that the NAS method can be used with distantly related, yet congeneric species references to successfully identify species having no reference available; we coin this approach as ‘phylogenetic capture.’ The broader implications of these findings is that NAS-based recovery of mtDNA sequences requires only a single mt genome from a given genus. It is also possible that by strategically selecting reference sequences for NAS that effectively span the phylogenetic distance of a given clade (i.e. basal and terminal clades, ≥ 1 members of a polytomy, 1 of 2 sister taxa, etc.) will enhance the discovery and identification of taxa wherein reference sequence data are absent or unavailable. We note that the NAS reference file for experiment C (*Ae. vexans*) included all publicly available mitogenomes on the NCBI organelle database at the time of our experiment (11,982 mitogenome sequences; accessed 16 Sept 2021). We observed no indication that the MinKNOW software or NAS method was impacted by this number of reference sequences. The implication of this observation is that a single mitogenome reference file spanning the tree of life can be used for the NAS phylogenetic capture method. If accurate, this approach has far-reaching implications for species barcoding and molecular systematics that extend beyond the scope of the analyses presented herein (e.g. pathogen discovery).

We were unable to recover blood-meal identifications for mosquitoes sampled in NAS experiments B (*Cx. territans, Cx. restuans*) and C (*Ae. vexans)*. In experiment B, one possible explanation for this observation is that blood meals may have been from a vertebrate source not included in our initial enrichment FASTA. For example, *Cx. territans* is known to feed from a variety of vertebrate sources, including reptiles and amphibians [57], groups which were not represented as enrichment references during this sequencing experiment. To overcome this potential limitation during subsequent experiments C and E, we elected to increase the number of representative mitogenomes and taxa in our references to increase the likelihood of positive blood meal-associated read recovery. As reviewed by Kent [3], there are several variables which either individually, or combined, could additionally influence successful blood-meal identification using molecular methods, including: (i) time between feeding event and sample preservation (longer times result in more host blood being digested); (ii) mammalian hosts having enucleated red blood cells (RBCs; successful identification dependent upon leukocytes present in a mammalian sample; increased chance of success if host has nucleated RBCs [e.g. birds, reptiles, amphibians]); and (iii) a high percentage of hematophagous insect DNA within extracts (bulk, whole individual DNA extracts result in majority of DNA originating from the hematophagous insect). Despite these observations, NAS for blood-meal identification has several advantages over traditional PCR approaches, in particular: (i) NAS is not confounded by PCR inhibitors (i.e. heme) present in blood [72]; and (ii) the method can detect multiple hosts within a heterogeneous blood meal when using a reference file of substantial phylogenetic breadth. Moreover, because nanopore sequencing is a single-molecule technology, NAS data negate the need for cloning and Sanger sequencing of mixed, multi-host PCR products that are the result of PCR primers targeting highly conserved regions.

Our NAS data consisted of enriched mtDNA sequences ranging from hundreds to thousands of nucleotide bases of a given sample, and downstream bioinformatic processes yielded de novo mitogenome assemblies for four insects for which no previous mitogenome existed (i.e. unavailable on public nucleotide repositories). The vast majority of available sequences for mt-based molecular barcoding consist of COI sequences (approx. 9.87 M) managed by the Barcode of Life Data System (BOLD; www.boldsystems.org). Thus, when combined with the BOLD database, NAS mitogenome sequencing is particularly useful for the molecular identification of insect vectors (as demonstrated above). Select mtDNA genes that are frequently used for species barcoding (i.e. genes encoding COI, cyt-b) are easily extracted from de novo assembled mitogenomes of hematophagous insects (and their corresponding blood meals), for rapid BLAST and phylogenetic analyses facilitated by the BOLD initiative. We anticipate that the availability of complete mitogenome assemblies will increase exponentially with the usage of single-molecule long-read sequencing methodologies (e.g., ONT and Pacific Biosciences [Menlo Park, CA, USA]). Reference mitogenomes of hematophagous insects will provide important insights into the evolutionary histories of disease vectors and will greatly assist with accurate taxonomic identification efforts of cryptic species.

## Conclusions

Nanopore sequencing through adaptive sampling is a revolutionary approach that can be leveraged to address many biological questions [37, 49, 50, 73–76]. Long-read single-molecule sequencing is ideally suited for recovering entire mitogenomes across the tree of life, thus opening the door to enhanced mt barcoding. Here, we demonstrate how NAS can be utilized to dually identify hematophagous insects and their blood-meal hosts through complete mitogenome sequencing and characterization of recovered mt barcoding genes. Our data indicate that NAS can generate sequence data enriched for mt reads over unenriched ‘traditional’ nanopore sequencing to improve downstream mitogenome assemblies. Importantly, the NAS-based strategies outlined here can be leveraged to sequence a wide diversity of arthropod taxa, as publicly available mitogenome references at the genus level are sufficient to capture mt reads for related species whose complete mitogenome has not yet been sequenced (i.e. ‘phylogenetic capture’). Given the well-documented ease of use and portability of ONT MinION instruments [34, 77, 78], we envision that similar NAS-based mt barcoding efforts, in addition to metagenomic pathogen surveillance, can be performed within individual research laboratories, including field-based locations, worldwide. Thus, we posit that NAS-based surveillance of hematophagous insects will greatly advance global One Health research projects and biosurveillance initiatives.

## Supplementary Information

**Additional file 1:** **Table S1.** Vector specimens examined for each sequencing experiment. SRA accession number includes raw FASTQ files from each experiment. NCBI organelle accession numbers are only available for the four vector species that had fully assembled mitochondrial genomes generated in this study.

**Additional file 2:** **Table S2.** Genbank accession numbers that were included in the reference FASTA file used during NAS sequencing runs. Experiment A included the only publically available representative of the genus *Chrysops *(*C. silvifacies*), a species that is not native to the study region of North America; various potential vertebrate hosts and a whole genome assembly for the bacterial pathogen *Francisella tularensis *were also included as enrichment references. Experiment B contained related mosquito species, an assortment of possible bird and mammal blood meals and possible pathogen targets of interest. Experiment E contained closely related vector species and possible bird, mammal, reptile and amphibian blood meals. Experiment C is not included in this table as we used the NCBI RefSeq download for the entire mitochondrial genome database provided by NCBI (https://ftp.ncbi.nlm.nih.gov/refseq/release/mitochondrion/; accessed 16 Sept 2021).

**Additional file 3:** **Table S3.** Sequences were randomly subsampled starting at 9000 sequences. The resulting subsampled files were mapped to the mitogenome of *Culex* *pipiens* using Minimap2.

**Additional file 4:** **Figure S1.** Mitogenome map for the black deer fly, *Chrysops niger*, sequenced during nanopore adaptive sampling Experiment A. Orientation of gene transcription is denoted with arrows and A + T content across the mitogenome is depicted on the innermost circle in light gray. 

**Additional file 5: Figure S2.** Maximum likelihood phylogenies generated based on the COI gene for hematophagous insects sequenced with NAS. Phylogenies were generated using 1000 bootstrap replicates with statistically supported nodes (≥ 75 bootstrap value) depicted with an asterisk (*). Consensus sequences generated in the present study are denoted in red with their corresponding experimental run; comparable sequences obtained through the Barcode of Life Data System (BOLD; www.boldsystems.org). a Maximum likelihood tree for deer flies in the genus *Chrysops* b Maximum likelihood tree for *Aedes* mosquitoes c Maximum likelihood tree for *Culex* mosquitoes.

**Additional file 6:** **Table S4. **Sequences downloaded from GenBank (species and accession numbers) used in the phylogenies of blood meals sequenced from experiment E.

## Data Availability

All raw sequence data generated during the course of this study have been deposited within the NCBI SRA biorepository and are publicly available (Bioproject: PRJNA775614; Biosamples: SAMN22604479-SAMN22604483; SAMN22888850-SAMN22888851). Mitochondrial genome assemblies resulting from our research have been deposited at GenBank (NCBI Accession OL351547-OL351550, OL471015).

## Abbreviations

BLAST: Basic local alignment search tool
BOLD: Barcode of Life Data System
COI: Cytochrome* c* oxidase subunit 1
cyt-b: Cytochrome b
mt: Mitochondrial
NAS: Nanopore adaptive sampling
NUMT: Nuclear mitochondrial DNA segment
ONT: Oxford Nanopore Technologies Inc

## References

[1] Pérez de León AA, Mitchell RD, Watson DW (2020). Ectoparasites of cattle. Vet Clin North Am Food Animal Prac.

[2] Gómez-Díaz E, Figuerola J (2010). New perspectives in tracing vector-borne interaction networks. Trends Parasitol.

[3] Kent RJ (2009). Molecular methods for arthropod bloodmeal identification and applications to ecological and vector-borne disease studies. Mol Ecol Resour.

[4] Hardy JL, Houk EJ, Kramer LD, Reeves WC (1983). Intrinsic factors affecting vector competence of mosquitoes for arboviruses. Annu Rev Entomol.

[5] Bartholomay LC, Michel K (2018). Mosquito immunobiology: the intersection of vector health and vector competence. Annu Rev Entomol.

[6] Quick J, Grubaugh ND, Pullan ST, Claro IM, Smith AD, Gangavarapu K (2017). Multiplex PCR method for MinION and Illumina sequencing of Zika and other virus genomes directly from clinical samples. Nat Protoc.

[7] Naveca FG, Claro I, Giovanetti M, de Jesus JG, Xavier J, de IaniFC M (2019). Genomic, epidemiological and digital surveillance of Chikungunya virus in the Brazilian Amazon. PLoS Negl Trop Dis.

[8] Hill SC, de Souza R, Thézé J, Claro I, Aguiar RS, Abade L (2020). Genomic surveillance of Yellow Fever virus epizootic in São Paulo, Brazil, 2016–2018. PLoS Pathog.

[9] Giovanetti M, Faria NR, Lourenço J, Goes de Jesus J, Xavier J, Claro IM (2020). Genomic and epidemiological surveillance of Zika Virus in the Amazon region. Cell Reports.

[10] Beebe NW (2018). DNA barcoding mosquitoes: advice for potential prospectors. Parasitology.

[11] Jinbo U, Kato T, Ito M (2011). Current progress in DNA barcoding and future implications for entomology. Entomol Sci.

[12] Alcaide M, Rico C, Ruiz S, Soriguer R, Muñoz J, Figuerola J (2009). Disentangling Vector-Borne transmission networks: a universal DNA barcoding method to identify vertebrate hosts from arthropod bloodmeals. PLoS ONE.

[13] Mukabana WR, Takken W, Knols BGJ (2002). Analysis of arthropod bloodmeals using molecular genetic markers. Trends Parasitol.

[14] Reeves LE, Gillett-Kaufman JL, Kawahara AY, Kaufman PE (2018). Barcoding blood meals: new vertebrate-specific primer sets for assigning taxonomic identities to host DNA from mosquito blood meals. PLoS Negl Trop Dis.

[15] Townzen JS, Brower AVZ, Judd DD (2008). Identification of mosquito bloodmeals using mitochondrial *cytochrome oxidase subunit I* and *cytochrome b* gene sequences. Med Vet Entomol.

[16] Gissi C, Iannelli F, Pesole G (2008). Evolution of the mitochondrial genome of Metazoa as exemplified by comparison of congeneric species. Heredity.

[17] Boore JL (1999). Animal mitochondrial genomes. Nucleic Acids Res.

[18] Cameron SL (2014). Insect mitochondrial genomics: implications for evolution and phylogeny. Annu Rev Entomol.

[19] Crampton-Platt A, Timmermans MJTN, Gimmel ML, Kutty SN, Cockerill TD, Vun Khen C (2015). Soup to tree: the phylogeny of beetles inferred by mitochondrial metagenomics of a Bornean rainforest sample. Mol Biol Evol.

[20] Crampton-Platt A, Yu DW, Zhou X, Vogler AP (2016). Mitochondrial metagenomics: letting the genes out of the bottle. GigaSci.

[21] Romero PE, Weigand AM, Pfenninger M (2016). Positive selection on panpulmonate mitogenomes provide new clues on adaptations to terrestrial life. BMC Evol Biol.

[22] Timmermans MJTN, Dodsworth S, Culverwell CL, Bocak L, Ahrens D, Littlewood DTJ (2010). Why barcode? High-throughput multiplex sequencing of mitochondrial genomes for molecular systematics. Nucleic Acids Res.

[23] Wanner N, Larsen PA, McLain A, Faulk C (2021). The mitochondrial genome and epigenome of the golden lion Tamarin from fecal DNA using nanopore adaptive sequencing. BMC Genomics.

[24] Bradley RD, Baker RJ (2001). A Test of the genetic species concept: cytochrome-*b* sequences and mammals. J Mammal.

[25] Creedy TJ, Andújar C, Meramveliotakis E, Noguerales V, Overcast I, Papadopoulou A (2021). Coming of age for COI metabarcoding of whole organism community DNA: towards bioinformatic harmonisation. Mol Ecol Resour.

[26] Borland EM, Kading RC (2021). Modernizing the toolkit for arthropod bloodmeal identification. Insects.

[27] Molaei G, Andreadis TG, Armstrong PM, Bueno R, Dennett JA, Real SV (2007). Host feeding pattern of *Culex quinquefasciatus* (Diptera: Culicidae) and its role in transmission of West Nile virus in Harris County, Texas. Am J Trop Med Hyg.

[28] Santiago-Alarcon D, Havelka P, Schaefer HM, Segelbacher G (2012). Bloodmeal analysis reveals avian *Plasmodium* infections and broad host preferences of *Culicoides* (Diptera: Ceratopogonidae) vectors. PLoS ONE.

[29] Schnell IB, Thomsen PF, Wilkinson N, Rasmussen M, Jensen LRD, Willerslev E (2012). Screening mammal biodiversity using DNA from leeches. Curr Biol.

[30] Videvall E, Bensch S, Ander M, Chirico J, Sigvald R, Ignell R (2013). Molecular identification of bloodmeals and species composition in *Culicoides* biting midges. Med Vet Entomol.

[31] Costa FO, Carvalho GR (2010). New insights into molecular evolution: prospects from the barcode of life initiative (BOLI). Theory Biosci.

[32] Kieran TJ, Gottdenker NL, Varian CP, Saldaña A, Means N, Owens D (2017). Blood meal source characterization using Illumina sequencing in the Chagas disease vector *Rhodnius pallescen* (Hemiptera: Reduviidae) in Panama. J Med Entomol.

[33] Muturi EJ, Dunlap C, Tchouassi DP, Swanson J (2021). Next generation sequencing approach for simultaneous identification of mosquitoes and their blood-meal hosts. J Vector Ecol.

[34] Blanco MB, Greene LK, Rasambainarivo F, Toomey E, Williams RC, Andrianandrasana L (2020). Next-generation technologies applied to age-old challenges in Madagascar. Conserv Genet.

[35] Hoenen T, Groseth A, Rosenke K, Fischer RJ, Hoenen A, Judson SD (2016). Nanopore sequencing as a rapidly deployable ebola outbreak tool. Emerg Infect Dis.

[36] Jain M, Olsen HE, Paten B, Akeson M (2016). The Oxford Nanopore MinION: delivery of nanopore sequencing to the genomics community. Genome Biol.

[37] Kipp EJ, Lindsey LL, Khoo BS, Faulk C, Oliver JD, Larsen PA (2021). Enabling metagenomic surveillance for bacterial tick-borne pathogens using nanopore sequencing with adaptive sampling. bioRxiv.

[38] Payne A, Holmes N, Clarke T, Munro R, Debebe BJ, Loose M (2021). Readfish enables targeted nanopore sequencing of gigabase-sized genomes. Nat Biotechnol.

[39] Darsie RF, Ward RA (2005). Identification and geographical distribution of the mosquitoes of North America, north of Mexico.

[40] De Coster W, D’Hert S, Schultz DT, Cruts M, Van Broeckhoven C (2018). NanoPack: visualizing and processing long-read sequencing data. Bioinformatics.

[41] Li H (2018). Minimap2: pairwise alignment for nucleotide sequences. Bioinformatics.

[42] Donath A, Jühling F, Al-Arab M, Bernhart SH, Reinhardt F, Stadler PF (2019). Improved annotation of protein-coding genes boundaries in metazoan mitochondrial genomes. Nucleic Acids Res.

[43] Greiner S, Lehwark P, Bock R (2019). OrganellarGenomeDRAW (OGDRAW) version 1.3.1: expanded toolkit for the graphical visualization of organellar genomes. Nucleic Acids Res.

[44] Katoh K, Standley DM (2013). MAFFT multiple sequence alignment software version 7: improvements in performance and usability. Mol Biol Evol.

[45] Stamatakis A (2014). RAxML version 8: a tool for phylogenetic analysis and post-analysis of large phylogenies. Bioinformatics.

[46] Behura SK, Lobo NF, Haas B, deBruyn B, Lovin DD, Shumway MF (2011). Complete sequences of mitochondria genomes of *Aedes aegypti* and *Culex quinquefasciatus* and comparative analysis of mitochondrial DNA fragments inserted in the nuclear genomes. Insect Biochem Mol Biol.

[47] Luo Q-C, Hao Y-J, Meng F, Li T-J, Ding Y-R, Hua Y-Q (2016). The mitochondrial genomes of *Culex tritaeniorhynchus* and *Culex pipiens pallens* (Diptera: Culicidae) and comparison analysis with two other *Culex* species. Parasites Vectors.

[48] Wang K, Li X, Ding S, Wang N, Mao M, Wang M (2016). The complete mitochondrial genome of the *Atylotus miser* (Diptera: Tabanomorpha: Tabanidae), with mitochondrial genome phylogeny of lower Brachycera (Orthorrhapha). Gene.

[49] Gan M, Wu B, Yan G, Li G, Sun L, Lu G (2021). Combined nanopore adaptive sequencing and enzyme-based host depletion efficiently enriched microbial sequences and identified missing respiratory pathogens. BMC Genomics.

[50] Martin S, Heavens D, Lan Y, Horsfield S, Clark MD, Leggett RM (2022). Nanopore adaptive sampling: a tool for enrichment of low abundance species in metagenomic samples. Genome Biol.

[51] Hlaing T, Tun-Lin W, Somboon P, Socheat D, Setha T, Min S (2009). Mitochondrial pseudogenes in the nuclear genome of *Aedes aegypti* mosquitoes: implications for past and future population genetic studies. BMC Genet.

[52] Ding Y-R, Li B, Zhang Y-J, Mao Q-M, Chen B (2018). Complete mitogenome of *Anopheles sinensis* and mitochondrial insertion segments in the nuclear genomes of 19 mosquito species. PLoS ONE.

[53] Dunphy BM, Rowley WA, Bartholomay LC (2014). A taxonomic checklist of the mosquitoes of Iowa. J Am Mosq Control Assoc.

[54] Gendernalik A, Weger-Lucarelli J, Garcia Luna SM, Fauver JR, Rückert C, Murrieta RA (2017). American *Aedes vexans* mosquitoes are competent vectors of Zika Virus. Am J Trop Med Hyg.

[55] Ndiaye EH, Fall G, Gaye A, Bob NS, Talla C, Diagne CT (2016). Vector competence of *Aedes vexans* (Meigen), *Culex poicilipes* (Theobald) and *Cx. quinquefasciatus* say from senegal for West and East African lineages of Rift Valley fever virus. Parasit Vectors.

[56] Turell MJ, Dohm DJ, Sardelis MR, O’Guinn ML, Andreadis TG, Blow JA (2005). An update on the potential of North American mosquitoes (Diptera: Culicidae) to transmit West Nile virus. J Med Entomol.

[57] Molaei G, Andreadis TG, Armstrong PM, Diuk-Wasser M (2008). Host-feeding patterns of potential mosquito vectors in connecticut, USA: molecular analysis of bloodmeals from 23 species of *Aedes*, *Anopheles*, *Culex*, *Coquillettidia*, *Psorophora*, and *Uranotaenia*. J Med Entomol.

[58] Nasci RS (1984). Variations in the blood-feeding patterns of *Aedes vexans* and *Aedes trivittatus* (Diptera: Culicidae)1. J Med Entomol.

[59] Pinger RR, Rowley WA (1975). Host preferences of *Aedes trivittatus* (Diptera: Culicidae) in Central Iowa. Am J Trop Med Hyg.

[60] Tiawsirisup S, Platt KB, Evans RB, Rowley WA (2005). A comparision of West Nile virus transmission by *Ochlerotatus trivittatus* (COQ.), *Culex pipiens* (L.), and *Aedes albopictus* (Skuse). Vector-Borne Zoonotic Dis.

[61] Pinger RR (1982). Presumed *Dirofilaria immitis* infections in mosquitoes (Diptera: Culicidae) in Indiana, USA. J Med Entomol.

[62] Strickman D, Darsie RF (1988). The previously undetected presence of *Culex restuans* (Diptera: Culicidae) in Central America, with notes on identification. Mosquito Syst.

[63] Johnson BJ, Robson MG, Fonseca DM (2015). Unexpected spatiotemporal abundance of infected *Culex restuans* suggest a greater role as a West Nile virus vector for this native species. Infect Genet Evol.

[64] Monath TP, Tsai TF (1987). St. Louis encephalitis: lessons from the last decade. Am J Trop Med Hyg.

[65] Apperson CS, Harrison BA, Unnasch TR, Hassan HK, Irby WS, Savage HM (2002). Host-Feeding Habits of *Culex* and Other Mosquitoes (Diptera: Culicidae) in the Borough of Queens in New York City, with characters and techniques for identification of *Culex* Mosquitoes. J Med Entomol.

[66] Ebel GD, Rochlin I, Longacker J, Kramer LD (2005). *Culex restuans* (Diptera: Culicidae) relative abundance and vector competence for West Nile virus. J Med Entomol.

[67] Bartlett-Healy K, Crans W, Gaugler R (2008). Temporal and Spatial Synchrony of *Culex territans* (Diptera: Culicidae) with their Amphibian hosts. J Med Entomol.

[68] Desser SS, Hong H, Martin DS (1995). The life history, ultrastructure, and experimental transmission of *Hepatozoon catesbianae* n. comb, an apicomplexan parasite of the bullfrog, *Rana catesbeiana* and the mosquito, *Culex territans* in Algonquin Park Ontario. J Parasitol.

[69] Pechumann L (1973). The insects of Virginia: No. 6. Horse flies and deer flies of Virginia (Diptera :Tabanidae). Va Polytechnic Inst State Univ Res Div Bull.

[70] Weiner TJ, Hansens EJ (1975). Species and numbers of bloodsucking flies feeding on hogs and other animals in southern New Jersey. J New York Entomol Soc.

[71] Jellison WL (1950). Tularemia geographical distribution of “Deerfly Fever” and the biting fly* Chrysops discalis* Williston. Public Health Rep.

[72] Akane A, Matsubara K, Nakamura H, Takahashi S, Kimura K (1994). Identification of the heme compound copurified with deoxyribonucleic acid (DNA) from bloodstains, a major inhibitor of polymerase chain reaction (PCR) amplification. J Forensic Sci.

[73] Miller DE, Sulovari A, Wang T, Loucks H, Hoekzema K, Munson KM (2021). Targeted long-read sequencing identifies missing disease-causing variation. Am J Hum Genet.

[74] Marquet M, Zöllkau J, Pastuschek J, Viehweger A, Schleußner E, Makarewicz O (2022). Evaluation of microbiome enrichment and host DNA depletion in human vaginal samples using Oxford Nanopore’s adaptive sequencing. Sci Rep.

[75] Lin Y, Dai Y, Liu Y, Ren Z, Guo H, Li Z (2022). Rapid PCR-based nanopore adaptive sequencing improves sensitivity and timeliness of viral clinical detection and genome surveillance. Front Microbiol.

[76] Cheng H, Sun Y, Yang Q, Deng M, Yu Z, Zhu G, et al. A rapid bacterial pathogen and antimicrobial resistance diagnosis workflow using Oxford nanopore adaptive sequencing method. Briefings Bioinform. 2022;23:bbac453. 10.1093/bib/bbac453. 10.1093/bib/bbac453 36259361

[77] Maestri S, Cosentino E, Paterno M, Freitag H, Garces JM, Marcolungo L (2019). A rapid and accurate MinION-based workflow for tracking species biodiversity in the field. Genes.

[78] Pomerantz A, Peñafiel N, Arteaga A, Bustamante L, Pichardo F, Coloma LA (2018). Real-time DNA barcoding in a rainforest using nanopore sequencing: opportunities for rapid biodiversity assessments and local capacity building. GigaScience.

