# Supplementary material for: Nanopore adaptive sampling for targeted mitochondrial genome sequencing and bloodmeal identification in hematophagous insects
Source: Parasit Vectors. 2023 Feb 14;16:68. doi: 10.1186/s13071-023-05679-3 (PMC9930342; doi:10.1186/s13071-023-05679-3)
Supplement: Supplementary file 3 — Additional file 3: Table S3. Sequences were randomly subsampled starting at 9000 sequences. The resulting subsampled files were mapped to the mitogenome of Culex pipiens using Minimap2. [file 13071_2023_5679_MOESM3_ESM.docx]

**Table S3.** Sequences were randomly subsampled starting at 9,000 sequences. The resulting subsampled files were mapped to the mitogenome of *Culex* *pipiens* using minimap2.

|  | **Randomly subsampled number of sequences** | | | | |  |
| --- | --- | --- | --- | --- | --- | --- |
| **Sample** | **9,000** | **5,000** | **2,000** | **1,000** | **500** | **Avg Mapped (%)** |
| D: *Culex restuans* | 172 | 96 | 34 | 23 | 12 | 2.04 |
| B: *Culex restuans* | 6,747 | 3,723 | 1,496 | 737 | 366 | 74.2 |
| B: *Culex territans* | 5,875 | 3,252 | 1,301 | 655 | 336 | 65.6 |
