# Supplementary figures and images for: Nanopore adaptive sampling for targeted mitochondrial genome sequencing and bloodmeal identification in hematophagous insects
Source: Parasit Vectors. 2023 Feb 14;16:68. doi: 10.1186/s13071-023-05679-3 (PMC9930342; doi:10.1186/s13071-023-05679-3)

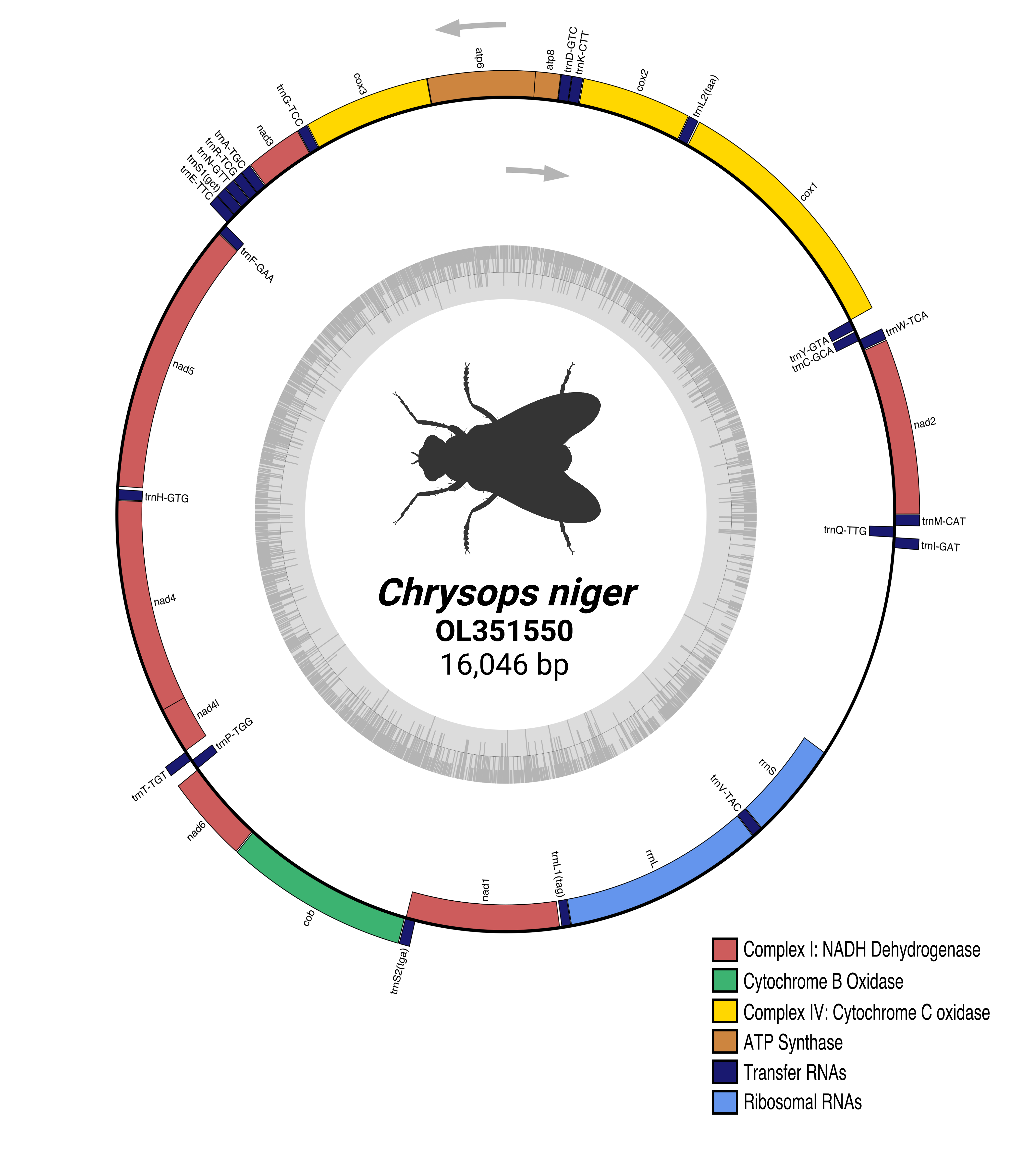

Supplement: Supplementary file 4 — Additional file 4: Figure S1. Mitogenome map for the black deer fly, Chrysops niger, sequenced during nanopore adaptive sampling Experiment A. Orientation of gene transcription is denoted with arrows and A + T content across the mitogenome is depicted on the innermost circle in light gray. [file 13071_2023_5679_MOESM5_ESM.png]

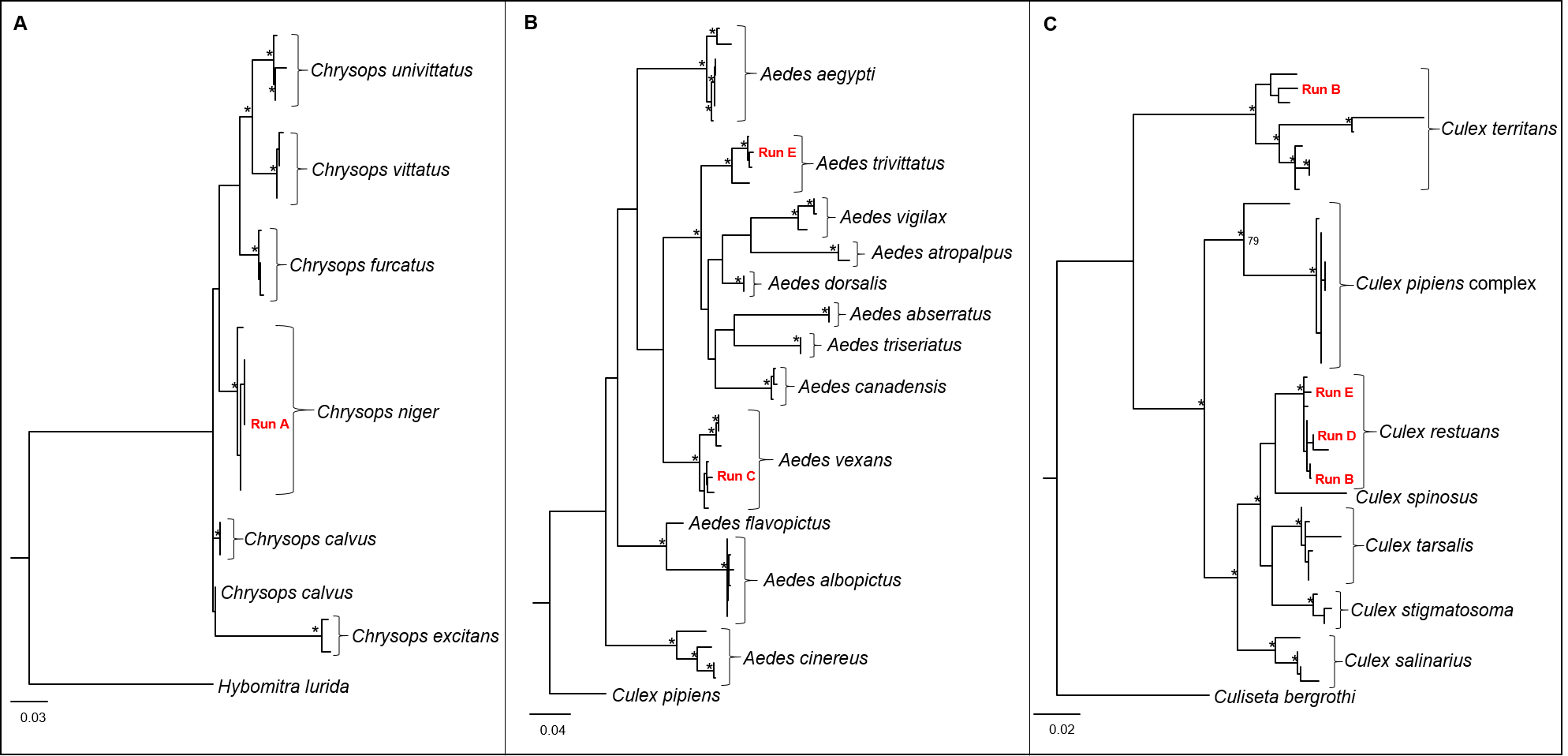

Supplement: Supplementary file 5 — Additional file 5: Figure S2. Maximum likelihood phylogenies generated based on the COI gene for hematophagous insects sequenced with NAS. Phylogenies were generated using 1000 bootstrap replicates with statistically supported nodes (≥ 75 bootstrap value) depicted with an asterisk (*). Consensus sequences generated in the present study are denoted in red with their corresponding experimental run; comparable sequences obtained through the Barcode of Life Data System (BOLD; www.boldsystems.org). a Maximum likelihood tree for deer flies in the genus Chrysops b Maximum likelihood tree for Aedes mosquitoes c Maximum likelihood tree for Culex mosquitoes. [file 13071_2023_5679_MOESM6_ESM.png]
