# Supplementary material for: Nanopore adaptive sampling for targeted mitochondrial genome sequencing and bloodmeal identification in hematophagous insects
Source: Parasit Vectors. 2023 Feb 14;16:68. doi: 10.1186/s13071-023-05679-3 (PMC9930342; doi:10.1186/s13071-023-05679-3)
Supplement: Supplementary file 6 — Additional file 6: Table S4. Sequences downloaded from GenBank (species and accession numbers) used in the phylogenies of blood meals sequenced from experiment E. [file 13071_2023_5679_MOESM4_ESM.docx]

**Table S4.** Sequences downloaded from GenBank (species and accession numbers) used in the phylogenies of bloodmeals sequenced from Exp. E.

| **House Sparrow** | **Cottontail** |
| --- | --- |
| *Passer domesticus:* MN356394.1, KM078784.1, AF407128.1 | *Sylvilagus floridanus:* AY012126.1, KU057246.1 |
| *Passer montanus:* MH211396.1, KM577704.1 | *Sylvilagus audobonii:* U67285.1 |
| *Passer ammodendri*: KT895996.1 | *Sylvilagus nuttallii*: KU057255.1 |
| *Montifringilla taczanowskii:* KJ148631.1 | *Lepus californicus:* KJ397614.1 |
| -- | *Lepus americanus:* KJ397613.1 |
